# Spatially varying effects of measured confounding variables on disease risk

**DOI:** 10.1186/s12942-021-00298-6

**Published:** 2021-11-11

**Authors:** Chih-Chieh Wu, Yun-Hsuan Chu, Sanjay Shete, Chien-Hsiun Chen

**Affiliations:** 1 Department of Environmental and Occupational Health, College of Medicine, National Cheng Kung University, 1 University Road, Tainan, 701 Taiwan; 2 Department of Statistics, College of Management, National Cheng Kung University, Tainan, Taiwan; 3 Department of Biostatistics, The University of Texas MD Anderson Cancer Center, Houston, TX USA; 4 Institute of Biomedical Sciences, Academia Sinica, Taipei, Taiwan

**Keywords:** Disease cluster, Hierarchical disease cluster, Spatial association, Spatial scan statistic, Spatially varying, Sudden infant death syndrome

## Abstract

**Background:**

The presence of considerable spatial variability in incidence intensity suggests that risk factors are unevenly distributed in space and influence the geographical disease incidence distribution and pattern. As most human common diseases that challenge investigators are complex traits and as more factors associated with increased risk are discovered, statistical spatial models are needed that investigate geographical variability in the association between disease incidence and confounding variables and evaluate spatially varying effects on disease risk related to known or suspected risk factors. Information on geography that we focus on is geographical disease clusters of peak incidence and paucity of incidence.

**Methods:**

We proposed and illustrated a statistical spatial model that incorporates information on known or hypothesized risk factors, previously detected geographical disease clusters of peak incidence and paucity of incidence, and their interactions as covariates into the framework of interaction regression models. The spatial scan statistic and the generalized map-based pattern recognition procedure that we recently developed were both considered for geographical disease cluster detection. The Freeman-Tukey transformation was applied to improve normality of distribution and approximately stabilize the variance in the model. We exemplified the proposed method by analyzing data on the spatial occurrence of sudden infant death syndrome (SIDS) with confounding variables of race and gender in North Carolina.

**Results:**

The analysis revealed the presence of spatial variability in the association between SIDS incidence and race. We differentiated spatial effects of race on SIDS incidence among previously detected geographical disease clusters of peak incidence and incidence paucity and areas outside the geographical disease clusters, determined by the spatial scan statistic and the generalized map-based pattern recognition procedure. Our analysis showed the absence of spatial association between SIDS incidence and gender.

**Conclusion:**

The application to the SIDS incidence data demonstrates the ability of our proposed model to estimate spatially varying associations between disease incidence and confounding variables and distinguish spatially related risk factors from spatially constant ones, providing valuable inference for targeted environmental and epidemiological surveillance and management, risk stratification, and thorough etiologic studies of disease.

## Introduction

The presence of considerable spatial variability with respect to incidence intensity of disease suggests that risk factors are unevenly distributed in space and influence the geographical disease incidence distribution and pattern. The detection and characterization of spatial, temporal, and space–time clusters of adverse health events aim for a greater understanding of the etiology and underlying causal mechanism of disease or the identification of common causal exposure for disease [1–4]. Although extensive spatial disease cluster detection analyses for numerous diseases have been performed, statistical spatial models that focus on quantitatively differentiating spatial effects of measured confounding variables on disease risk across the regions under study have not been fully explored. When increasing risk factors for adverse health events are detected or identified and could make a major impact on disease risk, robust methods that accurately estimate the spatially varying disease risk attributable to measured confounding variables are needed.

The vast majority of human common diseases that continue to challenge investigators are complex traits, such as cardiovascular disease, cancers, psychiatric disorders, and auto-immune disorders. They are caused by several or many genetic, environmental, or lifestyle factors and possibly interaction between risk factors combined with small effect each [5]. It is unlikely that one single risk factor or exposure for human complex disease can largely account for geographical heterogeneous distribution and clustering pattern of incidence. Even if the relative risk for specific factor and disease is high, we often note that not all occurrence of disease clustering is due only to specific exposure in question. Thus, development of statistical spatial model is needed that determines and assesses spatially varying associations between disease incidence and known or suspected risk factors.

The purpose of this paper is to propose and illustrate a statistical spatial model that quantitatively assesses the spatially varying effects of measured confounding variables that contribute to the observed spatial heterogeneity and clusters in disease incidence. The method is structured to precisely model measured confounding variables for spatially related risk factors fitted to previously detected geographical disease clusters of peak incidence and paucity of incidence and simultaneously evaluate the differential spatial effects of individual risk factors and possibly their interactions. It incorporates information on known or hypothesized risk factors, previously detected geographical disease clusters of peak incidence and paucity of incidence, and their interactions as covariates into the framework of interaction regression models with linear effects. The method is designed to estimate the spatially varying risk in incidence attributable to measured confounding variables in previously detected geographical disease clusters of peak incidence and incidence paucity and areas outside the geographical disease clusters. The Freeman-Tukey square-root transformation was applied to improve normality of distribution and approximately stabilize the variance in interaction regression models [6].

The spatial scan statistic and the generalized map-based pattern recognition procedure were both considered for geographical disease cluster detection in this report. The spatial scan statistic is widely used and has been extended to a variety of models for detecting spatial, temporal, and space–time clusters, retrospectively or prospectively [7]. The generalized map-based pattern recognition procedure that we recently developed is designed to recognize and construct hierarchical (in intensity) disease clusters of respectively high-risk areas and low-risk areas within close geographic proximity or contiguity on a map [8]. The spatial scan statistic and the generalized map-based pattern recognition procedure are used to detect geographically neighboring areas of peak incidence as well as incidence paucity in a spatial point process in general and allow for confounding variables.

In order to comprehensively characterize spatial variability with respect to incidence intensity, we proposed to use distinct spatial covariates for previously detected geographical disease clusters of peak incidence and for those of incidence paucity in the models. In comparison, most existing statistical methods and epidemiologic studies generally focus on large or peak incidence alone. In epidemiology, the occurrence of disease aggregations may be associated with risk factors of the disease. While an occurrence of unusually sparse incidence of disease may be due to the presence of protective factors or the absence of risk factors. We previously proposed and formulated statistical methods that focus on an unusually low incidence of disease in a unit of time in a discrete time series and in a spatial unit over space. We showed that statistical methods that are sensitive to incidence paucity in time or over space characterize opposite aspects of an observed incidence pattern and can be as meaningful and useful in epidemiology as the methods that focus on incidence clustering in our previous reports [9, 10].

We illustrated and exemplified proposed statistical spatial model by an analysis of incidence data on the spatial occurrence of sudden infant death syndrome (SIDS) incidence in North Carolina counties over the 4-year period in 1974–1978. Two possible confounding variables for SIDS are race and gender. The associations of SIDS incidence with race or gender have seen noted in the statistical and epidemiologic literature [7, 8, 11–15]. Information on spatial distribution of the race-specific and gender-specific live births is available in the literature [16]. The spatial risk analysis performed by our proposed statistical model well characterized and evaluated the spatially varying risk of SIDS incidence related to race and gender.

The statistical spatial model that we propose for spatial risk analysis addresses important problems. In particular:

1. We determine the presence or absence of geographical variability in the association between adverse health events and confounding variables.
2. We estimate the spatially varying risk in disease incidence attributable to measured confounding variables in previously detected geographical disease clusters of peak incidence and incidence paucity and areas outside the geographical disease clusters.
3. Without restrict ourselves to focusing on peak or large incidence, we incorporate spatial information on geographically neighboring areas with the highest and lowest incidence anomalies into the modeling.

Our proposed model is useful for spatial risk analysis in which measured confounding variables are observed and geographical disease clusters of peak incidence and paucity of incidence are determined. Confounding variables that contribute to spatial variation in risk of disease can include characteristics of various environmental exposures or characteristics of the study population. Our proposed model for spatial risk analysis provides valuable inference for targeted environmental and epidemiological surveillance and management, risk stratification, and thorough etiologic studies of disease.

## Methods

In this section, we introduce our statistical spatial model for assessing differential spatial effects of measured confounding variables, accounting for spatially heterogeneous distributions of disease of interest with respect to incidence intensity. We focus on geographical difference in risk related to measured confounding variables among geographical disease clusters of peak incidence and paucity of incidence and outside the geographical disease clusters. The data on spatial occurrence of SIDS in North Carolina counties provide an opportunity to illustrate the applications of our statistical spatial model for spatial risk analysis.

### Study population

SIDS is a subset of sudden unexpected infant death and remains the leading cause of death in infants aged from 1 month to 1 year in the United States, with more than 1900 deaths annually. The exact cause of SIDS is unknown, but it has long been believed to be multi-factorial in origin. The frequency of SIDS appears to be influenced by social, economic, and cultural factors, such as maternal education, race or ethnicity, and poverty. Racial disparity in infants who died of SIDS has persisted. The rate of SIDS in non-Hispanic African American infants and American Indian/Alaskan Native infants remains more than twice that of non-Hispanic white infants in 2016. Boys remain more likely to die of SIDS than girls. Information on epidemiologic, physiologic, and genetic research combined is likely to be needed for determining predispositions and identifying trends [15].

**Fig. 1:**
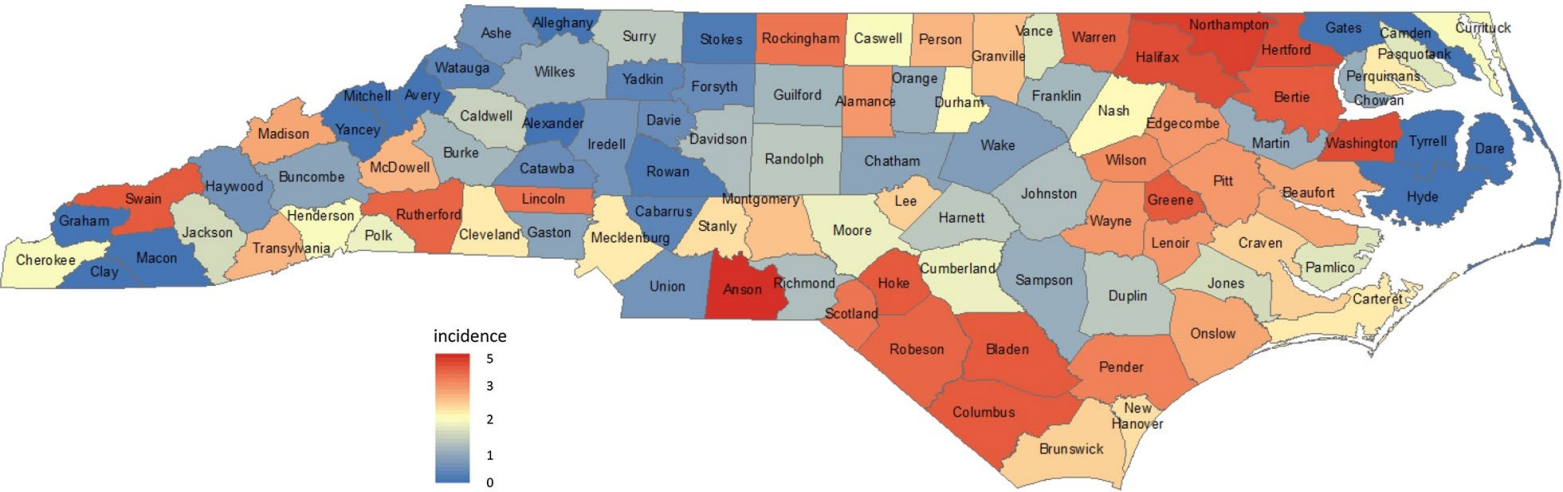
County-Specific SIDS Incidence Intensity Map in North Carolina with County Names

The data on spatial occurrence of SIDS patients with confounding variables of race and gender in North Carolina counties over the 4-year period, from July 01, 1974 to June 30, 1978, were used for illustrating the application of the statistical spatial model that we propose for spatial risk analysis. The information contained in this data set includes the number of SIDS patients and the number of live births by race and gender for each of the 100 counties of North Carolina during this period. The total number of live births was 329,962, in which the numbers of white male, white female, non-white male, and non-white female live births were 115,641, 109,222, 53,393, and 51,706, respectively. The total number of SIDS patients was 670, in which the numbers of white male, white female, non-white male, and non-white female SIDS patients were 164, 106, 222, and 178, respectively. The state-wide incidence rate was 2.031 in deaths per 1000 live births. The overall incidence rates for the entire state by race were 1.201 for white children and 3.806 for non-white children; by gender, 2.284 for male children and 1.765 for female children; and by race and gender, 1.418 for white males, 0.971 for white females, 4.158 for non-white males, and 3.443 for non-white females per 1000 live births. The complete data and details of the data sources and collection methods have been described elsewhere [16]. Figure 1 presents the county-specific SIDS incidence intensity map on the 100 counties of North Carolina with county names.

### The interaction regression model with linear effects

Let *Y* be the dependent or response variable for a disease of interest; *X*_*1*_ denote a measured covariate for a known or hypothesized risk factor; and *X*_*2*_ and *X*_*3*_ be indicator variables for areas in previously detected geographical disease clusters of peak incidence and those in previously detected geographical disease clusters of paucity of incidence, respectively. By letting the covariate *X*_*1*_ depend on the spatial covariate *X*_*2*_ (or *X*_*3*_), we use the interaction covariate *X*_*1*_*X*_*2*_ (or *X*_*1*_*X*_*3*_), the product of *X*_*1*_ and *X*_*2*_ (or *X*_*1*_ and *X*_*3*_), which estimates the excess of disease risk related to measured covariate *X*_*1*_ in geographical disease clusters of peak incidence (or paucity of incidence) over areas outside the geographical disease clusters.

Define an interaction regression model with linear effects:

1 $$Y \, = \beta_{0} + \beta_{1} X_{1} + \, \beta_{2} X_{1} X_{2} + \, \beta_{3} X_{1} X_{3} + \varepsilon$$

where *β*_*0*_, *β*_*1*_, *β*_*2*_, and *β*_*3*_ are regression coefficients to be estimated, using the method of least squares, and *ε* is an error term.

In this application, the response variable mapped is the SIDS rate and the regions under study are the 100 counties of North Carolina. We define *Y* = 1000 × SIDS incidence rate; *X*_*1*_ = non-white or male live-birth rate; and *X*_*2*_ (or *X*_*3*_) = 1 for counties in previously detected geographical SIDS clusters of peak incidence (or incidence paucity), determined by the spatial scan statistic or the generalized map-based pattern recognition procedure, or *X*_*2*_ = 0 (or *X*_*3*_ = 0) otherwise in our scheme. The *β*_*1*_ indicates the change in mean response of 1000 × SIDS incidence rate per unit increase in non-white or male live-birth rate, after controlling for other covariates. The *β*_*2*_ and *β*_*3*_ indicate the excess of SIDS risk related to race or gender in geographical SIDS clusters of peak incidence and paucity of incidence, respectively, over counties outside the geographical SIDS clusters.

### The Freeman-Tukey square-root transformation

Counties with smaller number of live-births will have larger variances for their estimated incidence rates, and tend to show higher fluctuation in incidence rates from the true unknown rate. The numbers of live-births are vastly different from county to county in this data set, ranging from 248 to 21,588. The Freeman-Tukey square-root transformation is often used to improve normality of distribution and approximately stabilize the variance; in particular, when data come as counts. The transformed data conform more closely to Gaussian data with a variance that does not depend on the mean. The Freeman-Tukey transformation performs better than the regular square-root transformation [6, 17].

One form of the Freeman-Tukey square-root transformation previously proposed and used on the SIDS data by Cressie and Chan is shown as follows:

$$Y^{FT} =\sqrt{1000s/n}+\sqrt{1000(s+1)/n}$$

$$X^{FT}_{1} =\sqrt{1000w/n}+\sqrt{1000(w+1)/n}$$

where *s* = SIDS patient number in a county, *w* = non-white live-birth number in a county, and *n* = live-birth number in a county [13]. We used this form of the Freeman-Tukey transformation on variables *Y* and *X*_*1*_ in Eq. (1). That is, we used the Freeman-Tukey transformed SIDS incidence rate *Y*^*FT*^ and the Freeman-Tukey transformed non-white or male live-birth rate *X*^*FT*^_*1*_ in the interaction regression model as follows:

2 $$Y^{FT} = \, \beta_{0} + \, \beta_{1} X^{FT}_{1} + \, \beta_{2} X^{FT}_{1} X_{2} + \, \beta_{3} X^{FT}_{1} X_{3} + \, \varepsilon .$$

**Fig. 2:**
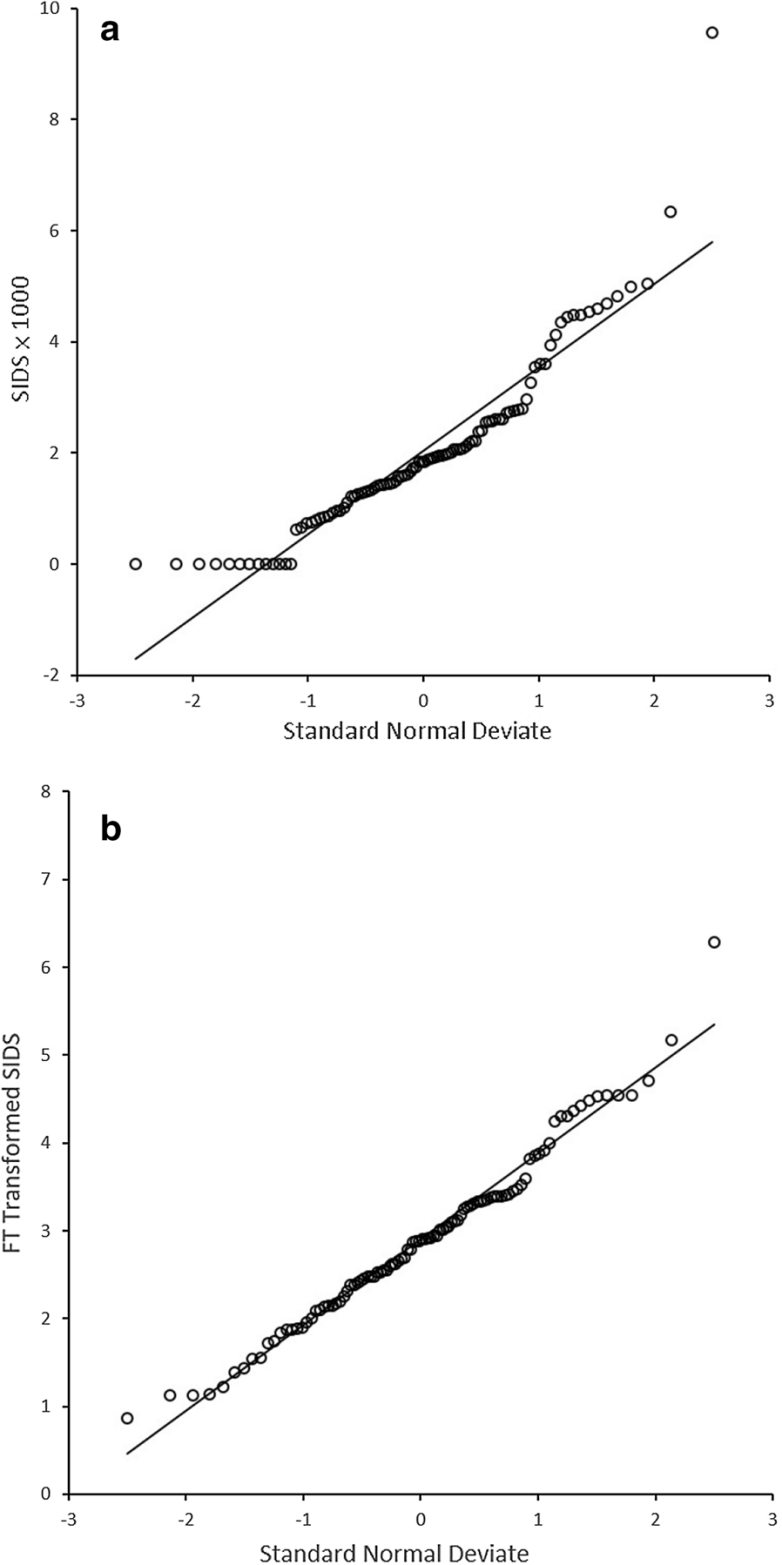
**A** Normal Probability Plot of SIDS Incidence Rates. **B** Normal Probability Plot of Freeman-Tukey Transformed SIDS Incidence Rates

The SIDS incidence rate *Y* and the Freeman-Tukey transformed SIDS incidence rate *Y*^*FT*^ are shown in a normal probability plot, presented in Fig. 2A and B, respectively. The *Y*^*FT*^ appears to much better conform to the assumption of normality. It is noted that the highest Freeman-Tukey transformed incidence rate of 6.28 in Fig. 2B was in Anson county whose raw incidence rate was 9.55 (= 15/1570), and there were 13 counties with 0 SIDS incidence but different numbers of live births, as shown in Fig. 2A. The generalization of Eq. (2) to more than one confounding variables is immediate. The threshold for statistical significance was set to 0.05 in this report.

### The spatial scan statistic

The spatial scan statistic searches for spatial disease clusters not explained by a baseline spatial point process without specifying their size or location a priori. It is able to identify the approximate location and range of the most likely disease clusters and secondary disease clusters and to perform a significance test for each cluster, based on the maximum likelihood ratio and using Monte Carlo hypothesis testing. The spatial scan statistic tends to detect relatively broad spatial clusters, and the detected most likely disease clusters may not be the regions with the highest rates. For example, in an analysis of SIDS incidence in North Carolina counties in 1974–1984, the spatial scan statistic identified the most likely disease cluster in the south with incidence of 3.821 and the secondary disease cluster in the northeast with incidence of 4.101 per 1000 live births, shown in Table 1 of the article by Kulldorff [7]. The state-wide incidence was 1.995 per 1000 live births.

**Table 1:** Summary of spatial SIDS cluster detection analysis by different models

Risk	Models	
	Generalized pattern recognition procedure	Spatial scan statistic
Higher rates	1.Northeast (6 counties: 5 Level-H1 and 1 Level-H2) with combined incidence of 4.98 2.South (6 counties: 1 Level-H1 and 5 Level-H2) with combined incidence of 4.06 3.Mid-East (6 counties: 1 Level-H1 and 5 Level-H3) with combined incidence of 3.09	1.Most likely disease cluster in the northeast (4 counties) with combined incidence of 5.12 2.Secondary disease cluster in the south (6 counties) with combined incidence of 3.76
Lower rates	1.Northwest (6 counties: 4 Level-L1 and 2 Level-L2) with combined incidence of 0.28 2.Mid-West (9 counties: 1 Level-L1 and 8 Level-L2) with combined incidence of 0.70 3.East (3 counties: 3 Level-L1) with combined incidence of 0.0	1.Most likely disease cluster in the mid-west (14 counties) with combined incidence of 1.10

The incidence rate in this table indicates the value of raw incidence per 1000 live births

The spatial scan statistic is widely used for spatial cluster detection analysis and allows for covariates. It has been extended to a variety of models for detecting spatial, temporal, and space–time clusters, retrospectively or prospectively, using ordinal, survival-time, multi-nominal, normal, and longitudinal data. Various models for the spatial scan statistic is implemented by the free program package of the SaTScan™ developed by Martin Kulldorff together with Information Management Services Inc (https://www.satscan.org/).

### The generalized map-based pattern recognition procedure

Cliff and Ord generalized an adjacency-based test statistic developed by Mantel [18] that measures spatial autocorrelation for binary data and uses the distribution of the number of adjacencies of geographic units [19]. When high-risk areas tend to be geographically adjacent to each other, the value of the test statistic tends to be large. The map-based pattern recognition procedure developed by Grimson et al. extends the utilities of the ordinary adjacency-based test statistic and is designed to determine hierarchical incidence intensity levels of mutually adjacent areas with the highest rates geographically. The procedure was also illustrated in an application to the SIDS data in North Carolina in 1974–1978 [20].

The map-based pattern recognition procedure incorporates information about the rank order of incidence intensity into the ordinary adjacency-based test statistic and constructs hierarchical incidence intensity patterns for some disease over geographical spaces by searching for hierarchical (in intensity) clusters of mutually adjacent areas with high rates. It prioritizes the areas with the highest rates in determining hierarchical incidence intensity levels of mutually adjacent areas with the highest rates geographically. The ordinary map-based pattern recognition procedure does not allow for covariates, exclusively focuses on peak incidence, and uses adjacency-based neighborhood system in determining the hierarchical incidence intensity levels. We previously used the map-based pattern recognition procedure to investigate the spatial clustering patterns of dengue outbreaks in Taiwan [21].

We recently generalized the ordinary map-based pattern recognition procedure in several important respects, including taking into account covariates that are known or hypothesized risk factors in the modeling, focusing on geographically neighboring areas of incidence paucity as well as peak incidence, and allowing for the use of distance-based neighborhood system in addition to the existing adjacency-based one in the definition of close geographical proximity [8]. The generalized pattern recognition procedure differentiates incidence intensity of geographical disease clusters of peak incidence and low incidence, adjusted for covariates that are known or hypothesized risk factors, as well as testing for the presence of clustering. The method is designed to recognize and construct hierarchical (in intensity) disease clusters of respectively high-risk areas and low-risk areas within close geographic proximity or contiguity on a map, including confounding variables as covariates.

Both the spatial scan statistic and the generalized map-based pattern recognition procedure are used to identify disease clustering or detect disease clusters in a spatial point process in general and allow for confounding variables. Because these 2 models are sensitive to different respects of spatially characteristic incidence clustering patterns and structured to provide different spatial clustering information, the geographical disease clusters detected by them are often different. We articulate the difference in sensitivity, applicability, and characteristics between these two models in our recent report [8].

## Results

In this section, we first present the analysis of detecting geographical disease clusters of peak incidence and incidence paucity performed by the generalized map-based pattern recognition procedure and the spatial scan statistic, respectively, based on data on the spatial occurrence of SIDS incidence in North Carolina counties. Secondly, we present the analysis of investigating geographical variability in the association between SIDS incidence and race and gender, using the proposed interaction regression model and the Freeman-Tukey square-root transformation.

### Geographical SIDS clusters by the generalized map-based pattern recognition procedure

The analysis of detecting geographical disease clusters of peak incidence and incidence paucity performed by the generalized map-based pattern recognition procedure was presented in our previous report [8]. We determined the 3 groups of counties to use in constructing hierarchical (in intensity) disease clusters of mutually neighboring high-risk counties with 3 different levels of intensity. Level-H1 counties are the 8 top ranking counties; Level-H2, 10 counties ranking from 9 to 18; Level-H3, 6 counties ranking from 19 to 24. The overall incidence of the 8 Level-H1, 10 Level-H2, and 6 Level-H3 counties combined are 5.57, 3.95, and 2.79 per 1000 live births, respectively. Correspondingly, We constructed 3 hierarchical intensity clusters of peak SIDS incidence that were located in the northeast (6 counties: 5 Level-H1 and 1 Level-H2) with combined incidence of 4.98, the south (6 counties: 1 Level-H1 and 5 Level-H2) with combined incidence of 4.06, and the mid-east (6 counties: 1 Level-H1 and 5 Level-H3) with combined incidence of 3.09 per 1000 live births.

**Fig. 3:**
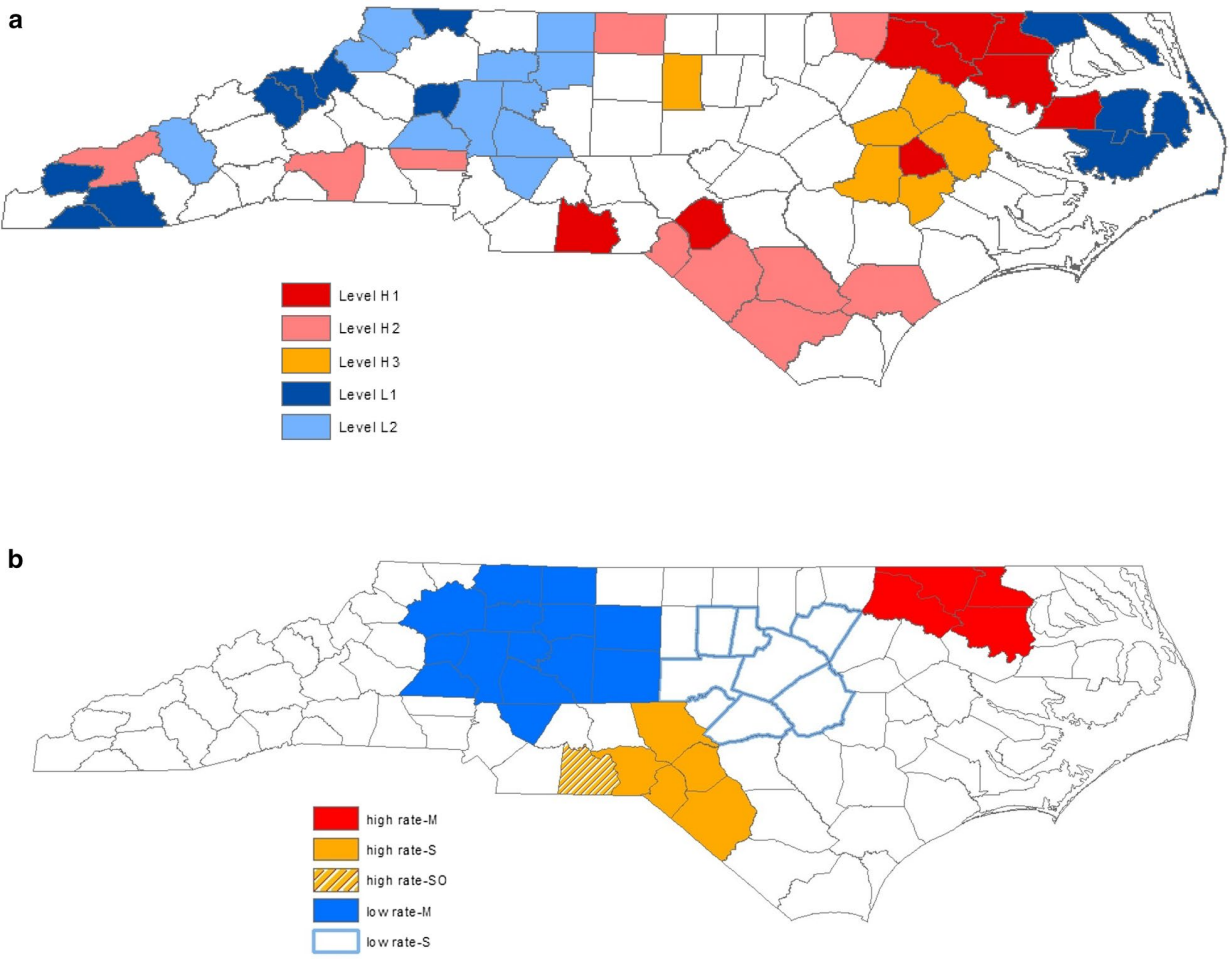
**A** SIDS Incidence Intensity-Level Map in North Carolina by Generalized Map-based Pattern Recognition Procedure. Fifty-two medium-risk counties are indicated in white that are not considered to be used in constructing hierarchical intensity clusters of peak and low SIDS incidence. **B** Most Likely and Secondary SIDS Clusters Map in North Carolina by Spatial Scan Statistic. Seventy-six counties are indicated in white that do not lie in the most likely and secondary disease clusters of peak SIDS incidence or the most likely disease cluster of low SIDS incidence

Next, we further constructed 3 hierarchical low-intensity clusters appearing in the northwest (6 counties: 4 Level-L1 and 2 Level-L2) with combined incidence of 0.28, the mid-west (9 counties: 1 Level-L1 and 8 Level-L2) with combined incidence of 0.70, and the eastern coast (3 counties: 3 Level-L1) with combined incidence of 0.00 per 1000 live births. Level-L1 counties are the 13 top ranking counties with 0 SIDS; Level-L2, 11 counties ranking from 87 to 77. The overall incidence of the 13 Level-L1 and 11 Level-L2 counties combined are 0 and 0.81 per 1000 live births, respectively. Figure 3A presents the county-specific SIDS incidence intensity-level map.

### Geographical SIDS clusters by the spatial scan statistic

We applied the Poisson model of the spatial scan statistic for detecting geographical disease clusters of peak incidence and incidence paucity to data on SIDS patients in North Carolina, using the program package of SaTScan™. The most likely disease cluster, denoted by M, and secondary disease cluster, denoted by S, of peak incidence were located in the northeast (4 counties in red) with a p-value of 1.11 × 10^–4^ and combined incidence of 5.12 and in the south (6 counties in yellow) with a p-value of 4.89 × 10^–4^ and combined incidence of 3.76 per 1000 live births, respectively, as shown in Fig. 3B. Anson county appeared as a highly significant sub-cluster inside the secondary cluster, denoted by SO, with a p-value of 6.06 × 10^–4^ and incidence of 9.55 per 1000 live births.

Next, we searched for geographical disease clusters of incidence paucity. The most likely disease cluster of low incidence, denoted by M, was located in the mid-west (14 counties in navy) with a p-value of 1.00 × 10^–6^ and combined incidence of 1.10 per 1000 live births. The secondary disease cluster of low incidence, denoted by S, in the mid-east (7 counties) was not statistically significant with a p-value of 6.12 × 10^–1^.

A summary of spatial SIDS cluster detection analysis based on the generalized map-based pattern recognition procedure and the spatial scan statistic is presented in Table 1. Note that the detected geographical SIDS clusters of high incidence in the article by Kulldorff were different from those identified and presented here because his analysis was based on a larger data of SIDS incidence in North Carolina, which were over the 9-year period in 1974–1984 [7]. In addition, his report did not search for spatial SIDS clusters of low incidence.

### Differential spatial effects of race

The expected incidence of SIDS patients, adjusted for race, in Anson was 4.35 per 1000 live births through indirect standardization, which was unacceptably low in comparison with its raw incidence of 9.55. We therefore removed Anson from the regression analysis to avoid one unusual value vastly affecting the fit to the other 99 North Carolina counties. Here, we applied the proposed interaction regression model, expressed in Eq. (2), to a total of 99 North Carolina counties for spatial risk analysis.

We started with a non-spatial analysis of SIDS incidence related to race by using the proposed model with no spatial covariates; that is, the linear regression model with one single covariate Race, denoted by *X*^*FT*^_*1*_, for Freeman-Tukey transformed non-white live-birth rate and *β*_*2*_ = *β*_*3*_ = 0. The covariate *X*^*FT*^_*1*_ was a highly significant predictor variable at a nominal significance level of 10^–3^ with the estimated coefficients *b*_*1*_ = 3.87 × 10^–2^, *se(b*_*1*_*)* = 5.53 × 10^–3^. The adjusted *R*^*2*^ for the *X*^*FT*^_*1*_-*Y*^*FT*^ regression line was 32.86% (*R*^*2*^ = 33.55%). The estimates of the model parameters are presented in the second column of Table 2.

**Table 2:** Summary of spatial risk analysis by different models with the generalized map-based pattern recognition procedure

Parameter	Covariates included in the models			
	Race	Race + Race × High-Risk	Race + Race × Low-Risk	Race + Race × High-Risk + Race × Low-Risk
*b*_*0*_	1.61 (1.95 × 10^–1^)	1.98 (1.83 × 10^–1^)	1.86 (1.79 × 10^–1^)	2.15 (1.66 × 10^–1^)
Covariate				
Race	3.87 × 10^–2^ (5.53 × 10^–3^)	2.11 × 10^–2^ (5.80 × 10^–3^)	3.54 × 10^–2^ (4.95 × 10^–3^)	2.02 × 10^–2^ (5.16 × 10^–3^)
Race × High-Risk		2.36 × 10^–2^ (4.26 × 10^–3^)		2.09 × 10^–2^ (3.82 × 10^–3^)
Race × Low-Risk			− 3.73 × 10^–2^ (7.14 × 10^–3^)	− 3.26 × 10^–2^ (6.32 × 10^–3^)
*F* for Overall Regression				
Regression | b_0_	48.97	47.23	44.71	48.71
Sequential *F*-Test				
Due to b_1_ | b_0_	48.97	63.90	62.21	80.89
Due to b_2_ | b_1_, b_0_		30.57		38.70
Due to b_3_ | b_2_, b_1_, b_0_			27.21	26.53
Adjusted *R*^*2*^ (*R*^*2*^)	32.86% (33.55%)	48.55% (49.60%)	47.15% (48.23%)	59.36% (60.60%)

1.Covariate High-Risk is coded as 1 for 18 counties in the 3 hierarchical intensity clusters of peak incidence and 0 otherwise

2.Covariate Low-Risk is coded as 1 for 18 counties in the 3 hierarchical intensity clusters of incidence paucity and 0 otherwise

Because different geographical SIDS clusters of peak incidence and incidence paucity were detected by the generalized map-based pattern recognition procedure and the spatial scan statistic, separate spatial risk analyses were performed and presented. In addition, measured spatial covariates to adjust for the counties in previously detected geographical SIDS clusters identified by these 2 models were coded accordingly.

### Spatial risk analysis with the generalized map-based pattern recognition

We tested the significance of geographical difference on disease risk in a measured covariate of race by letting the covariate *X*^*FT*^_*1*_ depend on the measured spatial covariate *X*_*2*_. That is, the interaction covariate *X*^*FT*^_*1*_*X*_*2*_, the product of *X*^*FT*^_*1*_ and *X*_*2*_, was used to estimate the excess of SIDS risk related to measured Freeman-Tukey transformed non-white live-birth rate in previously detected geographical SIDS clusters of peak incidence over counties outside these geographical SIDS clusters. Note that *X*_*2*_ is coded as 1 for 18 counties in the 3 hierarchical intensity clusters of peak incidence and 0 otherwise. Based on the proposed interaction regression model and *β*_*3*_ = 0, *F*(Regression | b_0_) = 47.23 (> *F*(2, 96, 0.999) = 7.43) was significant at a nominal significance level of 10^–3^ by the *F*-test for overall regression. The contribution of *X*^*FT*^_*1*_ and the additional contribution of *X*^*FT*^_*1*_*X*_*2*_ given that *X*^*FT*^_*1*_ was already introduced to the model were both very important and significant with *F*(due to b_1_ | b_0_) = 63.90 and *F*(due to b_2_ | b_1_, b_0_) = 30.57 (> *F*(1, 96, 0.999) = 11.52) by the sequential *F*-test. The estimates of the model parameters are presented in the third column of Table 2, including the adjusted *R*^*2*^ = 48.55% (*R*^*2*^ = 49.60%).

Next, we applied the proposed interaction regression model with *β*_*2*_ = 0 and used the interaction covariate *X*^*FT*^_*1*_*X*_*3*_ to estimate the excess of SIDS risk related to race in previously detected geographical SIDS clusters of incidence paucity over counties outside these geographical SIDS clusters. *X*_*3*_ is coded as 1 for 18 counties in the 3 hierarchical intensity clusters of incidence paucity and 0 otherwise. In this analysis, *X*^*FT*^_*1*_ and *X*^*FT*^_*1*_*X*_*3*_ after *X*^*FT*^_*1*_ was already in the equation were both highly significant with *F*(due to b_1_ | b_0_) = 62.21 and *F*(due to b_3_ | b_1_, b_0_) = 27.21 (> *F*(1, 96, 0.999) = 11.52). The *F*-test for overall regression was highly significant with *F*(Regression | b_0_) = 44.71 (> *F*(2, 96, 0.999) = 7.43). The result of the model with covariates *X*^*FT*^_*1*_ and *X*^*FT*^_*1*_*X*_*3*_ is presented in the fourth column of Table 2 with the adjusted *R*^*2*^ = 47.15% (*R*^*2*^ = 48.23%).

We further included both interaction covariates *X*^*FT*^_*1*_*X*_*2*_ and *X*^*FT*^_*1*_*X*_*3*_ in the model in the presence of the main effect of *X*^*FT*^_*1*_. Importantly, we found that the additional contributions of *X*^*FT*^_*1*_*X*_*2*_ given that *X*^*FT*^_*1*_ was already in the equation and *X*^*FT*^_*1*_*X*_*3*_ given that *X*^*FT*^_*1*_ and *X*^*FT*^_*1*_*X*_*2*_ were both in the equation remained highly significant each with *F*(due to b_2_ | b_1_, b_0_) = 38.70 and *F*(due to b_3_ | b_2_, b_1_, b_0_) = 26.53 (> *F*(1, 95, 0.999) = 11.53) by the sequential *F*-test. The *X*^*FT*^_*1*_ remained very important with *F*(due to b_1_ | b_0_) = 80.89 (> *F*(1, 95, 0.999) = 11.53). It is noted that *F*(Regression | b_0_) = 48.71 (> *F*(3, 95, 0.999) = 5.88) by the *F*-test for overall regression; *b*_*1*_ = 2.02 × 10^–2^, *se(b*_*1*_*)* = 5.16 × 10^–3^; *b*_*2*_ = 2.09 × 10^–2^, *se(b*_*2*_*)* = 3.82 × 10^–3^; *b*_*3*_ = − 3.26 × 10^–2^, *se(b*_*3*_*)* = 6.32 × 10^–3^; and the adjusted *R*^*2*^ = 59.36% (*R*^*2*^ = 60.60%). Each of the 3 predictor variables, *X*^*FT*^_*1*_, *X*^*FT*^_*1*_*X*_*2*_, and *X*^*FT*^_*1*_*X*_*3*_, was significant at a nominal significance level of 10^–3^ by the *t* test or partial *F-*test. The result of the model with covariates *X*^*FT*^_*1*_, *X*^*FT*^_*1*_*X*_*2*_, and *X*^*FT*^_*1*_*X*_*3*_ is shown in the fifth column of Table 2.

The inclusion of both the interaction covariates *X*^*FT*^_*1*_*X*_*2*_ and *X*^*FT*^_*1*_*X*_*3*_ to the proposed interaction regression model in the presence of the main effect of *X*^*FT*^_*1*_ was supported by the test statistics, although there existed a substantial correlation coefficient of 0.55 between *X*^*FT*^_*1*_ and *X*^*FT*^_*1*_*X*_*2*_, and a small correlation coefficient of − 0.13 between *X*^*FT*^_*1*_ and *X*^*FT*^_*1*_*X*_*3*_ in the model. It was further evidenced by the fact that the model with covariates *X*^*FT*^_*1*_, *X*^*FT*^_*1*_*X*_*2*_, and *X*^*FT*^_*1*_*X*_*3*_ had a substantially higher value of the adjusted *R*^*2*^ than that with *X*^*FT*^_*1*_ and *X*^*FT*^_*1*_*X*_*2*_ or that with *X*^*FT*^_*1*_ and *X*^*FT*^_*1*_*X*_*3*_ in comparison with the model with covariate *X*^*FT*^_*1*_ alone. Thus, our parsimonious fitted least-squares regression equation was

3 $$\widehat{{Y^{{FT}} }} = ~2.1528~ + ~0.0202~X^{{FT}} _{1} + ~0.0209~X^{{FT}} _{1} X_{2}~{-}~0.0326~X^{{FT}} _{1} X_{3}.$$

We classified as Region 1 the 63 counties outside the 6 geographical SIDS clusters of peak incidence and incidence paucity, the majority of which were medium-risk counties; as Region 2 the 18 counties in the 3 hierarchical intensity clusters of peak incidence (in the northeast, south, and mid-east); and as Region 3 the 18 counties in the 3 hierarchical intensity clusters of incidence paucity (in the northwest, mid-west, and eastern coast).

The coefficient *b*_*2*_ of *X*^*FT*^_*1*_*X*_*2*_ measures the differential effect of Freeman-Tukey transformed non-white live-birth rate *X*^*FT*^_*1*_ on the slope of the regression line between Region 1 and Region 2. The *b*_*2*_ = 0.0209 indicates that the slope of the regression line for Region 2 is higher by 0.0209 than that for Region 1. According to Eq. (3), the regression line has *Y*^*FT*^ slope 0.0202 for Region 1; *Y*^*FT*^ slope 0.0411 (= 0.0202 + 0.0209) for Region 2. Next, the coefficient *b*_*3*_ = − 0.0326 of *X*^*FT*^_*1*_*X*_*3*_ indicates that the slope of the regression line for Region 3 is lower by 0.0326 than that for Region 1; that is, the regression line has *Y*^*FT*^ slope − 0.0124 (= 0.0202 – 0.0326) for Region 3.

**Fig. 4:**
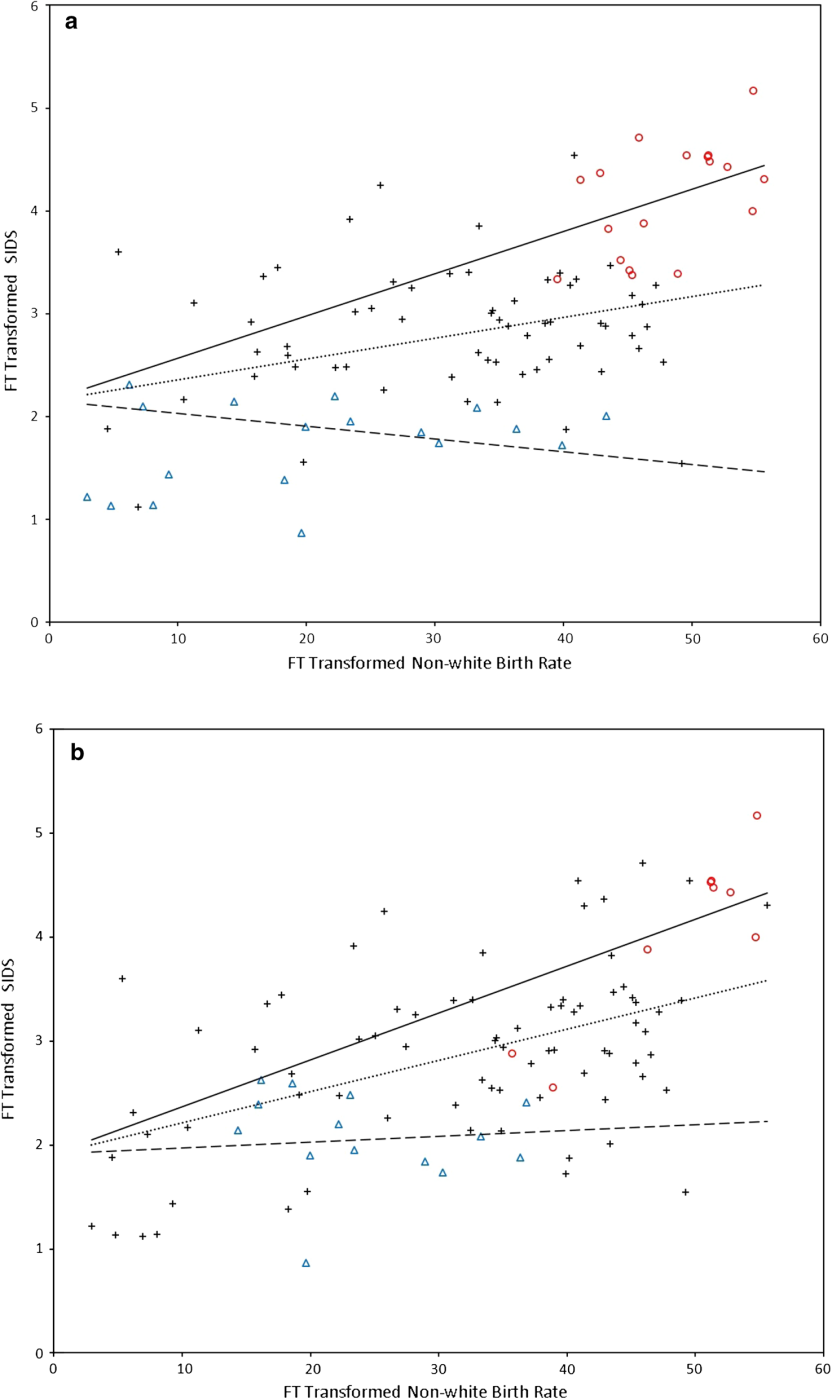
**A** Plot of Freeman-Tukey Transformed Non-White Live-Birth Proportion *X*^*FT*^_*1*_ versus Freeman-Tukey Transformed SIDS Incidence *Y*^*FT*^ for 99 North Carolina Counties and Fitted Regression Lines based on Generalized Map-based Pattern Recognition Procedure. Red Symbol  and Blue Symbol  Indicate Counties in Hierarchical Intensity Clusters of Peak Incidence and Incidence Paucity, Respectively. **B** Plot of Freeman-Tukey Transformed Non-White Live-Birth Proportion *X*^*FT*^_*1*_ versus Freeman-Tukey Transformed SIDS Incidence *Y*^*FT*^ for 99 North Carolina Counties and Fitted Regression Lines based on Spatial Scan Statistic. Red Symbol  and Blue Symbol  Indicate Counties in Likely SIDS Clusters of Peak Incidence and Incidence Paucity, Respectively

Letting the response function as a function of *X*^*FT*^_*1*_ conditional on *X*_*2*_ and *X*_*3*_, the spatial effect of race was highest in Region 2 with the response function equal to *2.1528* + *0.0411 X*^*FT*^_*1*_ for *X*_*2*_ = 1 and *X*_*3*_ = 0 and lowest in Region 3 with the response function = *2.1528—0.0124 X*^*FT*^_*1*_ for *X*_*2*_ = 0 and *X*_*3*_ = 1. The response function was *2.1528* + *0.0202 X*^*FT*^_*1*_ for Region 1 with *X*_*2*_ = 0 and *X*_*3*_ = 0. Figure 4A shows a plot of *X*^*FT*^_*1*_ versus *Y*^*FT*^ for the 99 North Carolina counties as well as the 3 fitted regression lines based on the generalized map-based pattern recognition procedure.

In conclusion, we determined the presence of spatial variability in the association between SIDS incidence and race and estimated the differential spatial effects of race on SIDS incidence among the 3 distinct regions defined by the generalized map-based pattern recognition procedure.

### Spatial risk analysis with the spatial scan statistic

We applied the proposed model in Eq. (2) to the geographical SIDS clusters of peak incidence and incidence paucity detected by the spatial scan statistic, as shown in Fig. 3B. In this application, *X*_*2*_ is coded as 1 for 9 counties in previously detected most likely disease cluster in the northeast with 4 counties and secondary disease cluster in the south with 5 counties of peak incidence and 0 otherwise. Note that the secondary disease cluster S comprises only 5 counties rather than 6 here because Anson is removed from this analysis.

Based on the proposed interaction regression model and *β*_*3*_ = 0, the contribution of covariate Race, denoted by *X*^*FT*^_*1*_, was significant at a nominal significance level of 10^–3^ with *F*(due to b_1_ | b_0_) = 52.66 (> *F*(1, 96, 0.999) = 11.52), and the additional contribution of *X*^*FT*^_*1*_*X*_*2*_ given that *X*^*FT*^_*1*_ was in the equation was significant at a nominal significance level of 10^–2^ with *F*(due to b_2_ | b_1_, b_0_) = 8.30 (> *F*(1, 96, 0.99) = 6.91) by the sequential *F*-test. The *F*-test for overall regression was highly significant with *F*(Regression | b_0_) = 30.48 (> *F*(2, 96, 0.999) = 7.43). The result of the model with covariates *X*^*FT*^_*1*_ and *X*^*FT*^_*1*_*X*_*2*_ is shown in the third column of Table 3, including the adjusted *R*^*2*^ = 37.56% (*R*^*2*^ = 38.84%).

**Table 3:** Summary of spatial risk analysis by different models with the spatial scan statistic

Parameter	Covariates included in the models			
	Race^1^	Race + Race × High-Risk	Race + Race × Low-Risk	Race + Race × High-Risk + Race × Low-Risk
*b*_*0*_	1.61 (1.95 × 10^–1^)	1.75 (1.94 × 10^–1^)	1.79 (1.96 × 10^–1^)	1.91 (1.94 × 10^–1^)
Covariate				
Race	3.87 × 10^–2^ (5.53 × 10^–3^)	3.22 × 10^–2^ (5.79 × 10^–3^)	3.59 × 10^–2^ (5.38 × 10^–3^)	3.00 × 10^–2^ (5.61 × 10^–3^)
Race × High-Risk		1.62 × 10^–2^ (5.63 × 10^–3^)		1.52 × 10^–2^ (5.41 × 10^–3^)
Race × Low-Risk			− 2.58 × 10^–2^ (8.30 × 10^–3^)	− 2.44 × 10^–2^ (8.03 × 10^–3^)
*F* for Overall Regression				
Regression | b_0_	48.97	30.48	31.48	25.13
Sequential *F*-Test				
Due to b_1_ | b_0_	48.97	52.66	53.33	57.17
Due to b_2_ | b_1_, b_0_		8.30		9.01
Due to b_3_ | b_2_, b_1_, b_0_			9.63	9.22
Adjusted *R*^*2*^ (*R*^*2*^)	32.86% (33.55%)	37.56% (38.84%)	38.35% (39.61%)	42.49% (44.25%)

1.The second column is identical to the second column of Table 2

2. Covariate High-Risk is coded as 1 for 9 counties in the most likely and secondary clusters of peak incidence and 0 otherwise

3. Covariate Low-Risk is coded as 1 for 14 counties in the most likely cluster of incidence paucity and 0 otherwise

With *X*_*3*_ coded as 1 for 14 counties in previously detected most likely disease cluster of incidence paucity and 0 otherwise, we next applied the proposed interaction regression model with *β*_*2*_ = 0. Note that the 7 counties in the secondary disease cluster of incidence paucity located in the mid-east are all coded as 0 as this cluster is not statistically significant at a nominal significance level of 0.05.

We found that the contribution of *X*^*FT*^_*1*_ was significant at a nominal significance level of 10^–3^ with *F*(due to b_1_ | b_0_) = 53.33 (> *F*(1, 96, 0.999) = 11.52) and the additional contribution of *X*^*FT*^_*1*_*X*_*3*_ given that *X*^*FT*^_*1*_ was in the equation was significant at a nominal significance level of 10^–2^ with *F*(due to b_3_ | b_1_, b_0_) = 9.63 (> *F*(1, 96, 0.99) = 6.91) by the sequential *F*-test. The *F*-test for overall regression remained highly significant with *F*(Regression | b_0_) = 31.48 (> *F*(2, 96, 0.999) = 7.43). The result of the model with *X*^*FT*^_*1*_ and *X*^*FT*^_*1*_*X*_*3*_ is presented in the fourth column of Table 3 with the adjusted *R*^*2*^ = 38.35% (*R*^*2*^ = 39.61%).

Incorporating covariates *X*^*FT*^_*1*_, *X*^*FT*^_*1*_*X*_*2*_, and *X*^*FT*^_*1*_*X*_*3*_ all into the proposed interaction regression model, we found that *X*^*FT*^_*1*_, *X*^*FT*^_*1*_*X*_*2*_ given that *X*^*FT*^_*1*_ was in the equation, and *X*^*FT*^_*1*_*X*_*3*_ given that both *X*^*FT*^_*1*_ and *X*^*FT*^_*1*_*X*_*2*_ were in the equation were all important and significant contributors to the observed spatial variation in SIDS risk each with *F*(due to b_1_ | b_0_) = 57.17 (> *F*(1, 95, 0.999) = 11.53), *F*(due to b_2_ | b_1_, b_0_) = 9.01, and *F*(due to b_3_ | b_2_, b_1_, b_0_) = 9.22 (> *F*(1, 95, 0.99) = 6.91). By the *F*-test for overall regression, *F*(Regression | b_0_) = 25.13 (> *F*(3, 95, 0.999) = 5.88) was highly significant. The estimates of the model parameters are presented in the fifth column of Table 3 with *b*_*1*_ = 3.00 × 10^–2^, *se(b*_*1*_*)* = 5.61 × 10^–3^; *b*_*2*_ = 1.52 × 10^–2^, *se(b*_*2*_*)* = 5.41 × 10^–3^; *b*_*3*_ = − 2.44 × 10^–2^, *se(b*_*3*_*)* = 8.03 × 10^–3^; and the adjusted *R*^*2*^ = 42.49% (*R*^*2*^ = 44.25%). The covariates *X*^*FT*^_*1*_, *X*^*FT*^_*1*_*X*_*2*_, and *X*^*FT*^_*1*_*X*_*3*_ were significant each at a nominal significance level of 10^–2^ by the *t* test or partial *F-*test.

Although the statistical evidence to include both *X*^*FT*^_*1*_*X*_*2*_ and *X*^*FT*^_*1*_*X*_*3*_ to the proposed interaction regression model in the presence of the main effect of *X*^*FT*^_*1*_ was not as strong as the previous application, we concluded the presence of spatially varying association between SIDS incidence and race. We found that the correlation coefficient between *X*^*FT*^_*1*_ and *X*^*FT*^_*1*_*X*_*2*_ = 0.39 remained substantial but smaller than the one (= 0.55) in the previous application. The correlation coefficient between *X*^*FT*^_*1*_ and *X*^*FT*^_*1*_*X*_*3*_ = − 0.17 was similar to the one (= − 0.13) in the previous application. The parsimonious fitted least-squares regression equation in this application was.

4 $$\widehat{{Y^{{FT}} }} = ~1.9133~ + ~0.0300~X^{{FT}} _{1} ~ + ~0.0152~X^{{FT}} _{1} X_{2}~{-}~0.0244~X^{{FT}} _{1} X_{3}.$$

We estimated the differential spatial effects of race on SIDS among the geographical SIDS clusters of incidence anomalies and outside the geographical SIDS clusters, detected by the spatial scan statistic. According to Eq. (4), the spatial effect of race was highest in the most likely and secondary disease clusters of peak incidence with the response function equal to *1.9133* + *0.0452 X*^*FT*^_*1*_ for *X*_*2*_ = 1 and *X*_*3*_ = 0 and lowest in the most likely disease cluster of incidence paucity with the response function = *1.9133* + *0.0056 X*^*FT*^_*1*_ for *X*_*2*_ = 0 and *X*_*3*_ = 1. The response function was *1.9133* + *0.0300 X*^*FT*^_*1*_ for 76 counties outside the detected geographical SIDS clusters by the spatial scan statistic with *X*_*2*_ = 0 and *X*_*3*_ = 0. Figure 4B shows a plot of *X*^*FT*^_*1*_ versus *Y*^*FT*^ for the 99 North Carolina counties as well as the 3 fitted regression lines based on the spatial scan statistic.

Table 4 gives a sample of counties with the observations used for the estimation of the parameters of the models expressed in Eqs. (3) and (4), respectively presented in the fifth column of Tables 2 and 3, as well as the fitted values and residuals.

**Table 4:** A sample of North Carolina counties with observations, fitted values, and residuals with full models

County			Generalized pattern recognition procedure				Spatial scan statistic			
	***Y***^***FT***^	***X***^***FT***^_***1***_	*X*_*2*_	*X*_*3*_	$\widehat{{Y}^{FT}}$	*Y*^*FT*^*-*$\widehat{{Y}^{FT}}$	*X*_*2*_	*X*_*3*_	$\widehat{{Y}^{FT}}$	*Y*^*FT*^*-*$\widehat{{Y}^{FT}}$
Alamance	3.399	32.629	0	0	2.812	0.587	0	0	2.892	0.507
Alexander	0.866	19.637	0	1	1.909	− 1.043	0	1	2.023	− 1.157
Alleghany	1.433	9.284	0	1	2.038	− 0.605	0	0	2.192	− 0.759
Ashe	2.311	6.203	0	1	2.076	0.236	0	0	2.099	0.212
Avery	1.132	4.793	0	1	2.093	− 0.962	0	0	2.057	− 0.926
Beaufort	3.336	41.003	0	0	2.981	0.355	0	0	3.143	0.193
Bertie	4.428	52.764	1	0	4.321	0.107	1	0	4.298	0.130
Bladen	4.366	42.863	1	0	3.915	0.452	0	0	3.199	1.167
Brunswick	2.527	34.778	0	0	2.855	− 0.328	0	0	2.957	− 0.430
Buncombe	2.474	22.255	0	0	2.602	− 0.129	0	0	2.581	− 0.107

### Spatial effects of gender

Gender was another important risk factor for SIDS incidence in this data. We found a significant difference between state-wide SIDS incidence rates for male children and female children, 2.284 versus 1.765 per 1000 live births, with a p-value of 1.07 × 10^–3^.

Letting covariate Gender, denoted by *X*^*FT*^_*1*_, for Freeman-Tukey transformed male live-birth rate and *β*_*2*_ = *β*_*3*_ = 0 in Eq. (2), the linear regression model with *Y*^*FT*^ indicated the non-significance of sex difference on SIDS risk in geography with the estimated coefficients *b*_*1*_ = 2.35 × 10^–1^, *se(b*_*1*_*)* = 1.89 × 10^–1^, which gives a p-value of 0.22 by the *t* test or partial *F-*test. It was further evidenced by the fact that the values of the adjusted *R*^*2*^ (< 0.6%), *R*^*2*^ (= 1.6%), and the correlation coefficient between *X*^*FT*^_*1*_ and *Y*^*FT*^ (= − 0.13) were all very low.

**Fig. 5:**
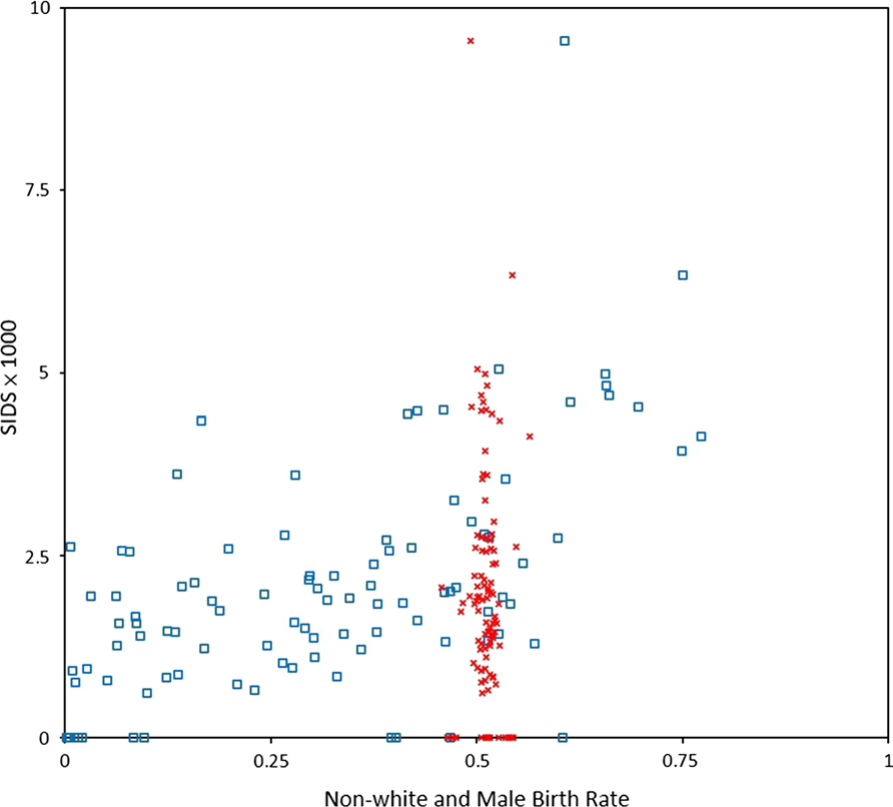
Plot of SIDS Incidence × 1000 versus Non-White (Blue Symbol  ) and Male (Red Symbol  ) Live-Birth Proportion

A plot of SIDS incidence × 1000 versus non-white and male live-birth rates for the 100 North Carolina counties, presented in Fig. 5, shows that non-white live-birth rate is highly spatially varying distributed, but male live-birth rate lies around 0.5. The result related to gender was very different from the previous one related to race because of the discrepancy between spatial distributions for race and gender. We concluded the absence of spatial association between SIDS incidence and gender. The spatial risk analysis of SIDS incidence performed by our proposed model that we have presented in this section well characterizes and assesses spatially varying associations between SIDS incidence and race and gender in studies of geographical disease clusters of peak incidence and paucity of incidence.

## Discussion

As more genetic, environmental, or lifestyle factors associated with increased disease risk are discovered, the paucity of statistical spatial models that accurately estimate the spatially varying disease risk attributable to the measured confounding variables and account for spatial heterogeneity and clustering in disease incidence becomes particularly pronounced.

In this paper, we have presented a general framework for differentiating geographical variability in disease risk related to measured confounding variables and assessing spatially varying associations between disease incidence and confounding variables. Information on geography that we focus on is geographical disease clusters of peak incidence and paucity of incidence identified by the spatial scan statistic and the generalized map-based pattern recognition procedure. We formulated an interaction regression model with linear effects by allowing for interaction covariates between a measured covariate for known or hypothesized risk factor and measured spatial covariates for previously detected geographical disease clusters of highest and lowest incidence anomalies to be tested for significance and accounted for in the model. We further proposed the use of the Freeman-Tukey transformation to improve normality of distribution and approximately stabilize the variance in the model. Our method aims for robust and reliable estimation of differential spatial effects on disease risk related to measured confounding variables for known or hypothesized risk factors among previously detected geographical disease clusters of peak incidence and paucity of incidence and areas outside the geographical disease clusters.

The detection of geographical disease clusters of highest and lowest incidence anomalies serves as a preliminary step that expedites subsequent investigation of disease etiology and spatial analysis of epidemicity. Both the spatial scan statistic and the generalized map-based pattern recognition procedure that we recently developed are designed to detect spatial disease clustering and allow for confounding variables, permitting the investigators to determine whether or not the previously detected geographical disease clusters of incidence anomalies can be explained by the covariates incorporated and to investigate other hidden spatially related risk factors if there still exist geographical disease clusters, after adjusting for known or hypothesized risk factors.

However, we are further interested in characterizing and evaluating to what extent the known or hypothesized risk factors contribute to the observed spatial heterogeneity and clustering in disease incidence and explain the observed spatial variability in risk of incidence across the regions under study. To what extent the risk factor explains spatial variability on disease risk estimation depends on several factors, including the degree of heterogeneity and complexity of the human complex disease. In addition, with information on estimates of disease risk attributable to known or hypothesized risk factors provided by the application of our proposed model, spatial effects of unknown risk factors will be simultaneously evaluated, leading to advance or generate studies of etiology of disease with unknown causes and the identification of hidden causal exposure for disease.

Statistical validity and sensitivity of the statistical spatial models proposed in this report are evidenced by our previously proposed methods for cancer risk analyses that are relevant to genetic, environmental, and epidemiological risk factors and determine their interactions in studies of familial clustering of cancer patients. These methods precisely model the measured genetic, environmental, and epidemiological risk factors for relatives in a family and incorporate this information into mathematical modeling in the framework of regressive logistic models [22] and Cox proportional hazards regression models [23, 24].

Equal nominal weights for counties in previously detected geographical disease clusters of peak incidence or incidence paucity are proposed in the modeling. It is possible to achieve higher power by the weighting schemes that assign different weights to the counties in respectively most likely and secondary disease clusters, determined by the spatial scan statistic, and to the counties in disease clusters according to the corresponding hierarchy in intensity, determined by the generalized map-based pattern recognition procedure. Further investigation into various weighting schemes is warranted in the future.

In this report, we illustrated and exemplified our proposed model by an analysis of incidence data on the spatial occurrence of SIDS in 100 North Carolina counties with 2 possible confounding variables of race and gender. The SIDS risk attributable to race is significantly higher in the 3 hierarchical intensity clusters of peak incidence and significantly lower in the 3 hierarchical intensity clusters of incidence paucity than the 63 counties outside these 6 geographical SIDS clusters, the majority of which were medium-risk counties.

Although the statistical evidence is not as strong, we differentiated the spatial effects of race on SIDS incidence, determined by the spatial scan statistic. The SIDS risk attributable to race is significantly higher in the most likely and secondary disease clusters of peak incidence and significantly lower in the most likely disease cluster of incidence paucity than the areas outside these geographical SIDS clusters. The covariate Race for Freeman-Tukey transformed non-white live-birth proportion serves as a proxy of important genetic, economic, or cultural factors, such as genetic predisposition, education level, and socioeconomic status. In addition, we found null spatial association between SIDS incidence and gender.

## Conclusion

The application to the data on North Carolina SIDS incidence illustrates and demonstrates the ability of our proposed interaction regression model to apply to geographical disease clusters determined by various spatial disease cluster detection models, distinguish spatially related risk factors from spatially constant ones, and estimate spatially varying associations between disease incidence and confounding variables. As Rothman and many others pointed out that we should not be aiming to detect clustering, but to understand why clusters occur [25, 26]. A powerful study design that focuses on extreme values has been proposed in gene mapping studies, in which geneticists collect a group of discordant sib pairs with extreme traits for detecting commonly shared genetic defects of a disease [27].

Accurate space-specific assessment of disease risk for known risk factors (race and gender in this application) would provide valuable inference for targeted environmental and epidemiological surveillance and management, risk stratification, and better risk prediction and prevention of disease incidence. In addition, spatial risk analysis performed by our proposed model provides a greater understanding of the effects of spatially related and spatially constant risk factors on disease incidence, which could ultimately lead to thorough etiologic studies of human complex disease.

## Data Availability

Information on data on the spatial occurrence of SIDS in North Carolina counties from July 01, 1974 to June 30, 1978 is available on the paper: Cressie N (1992). Smoothing regional maps using empirical Bayes predictors. *Geographical Analysis*, 24:75–95.

## Abbreviation

SIDS: Sudden infant death syndrome
